# Incidence, Management Experience and Characteristics of Patients with Giardiasis and Common Variable Immunodeficiency

**DOI:** 10.3390/jcm11237007

**Published:** 2022-11-27

**Authors:** Irene Díaz-Alberola, Juan Francisco Gutiérrez-Bautista, Andrea Espuch-Oliver, José María García-Aznar, Per Anderson, Pilar Jiménez, Carmen Hidalgo-Tenorio, Miguel Ángel López-Nevot

**Affiliations:** 1 Servicio de Análisis Clínicos e Inmunología, Hospital Universitario Virgen de las Nieves, 18014 Granada, Spain; 2 Programa de Doctorado en Biomedicina, University of Granada, 10816 Granada, Spain; 3 Instituto de Investigación Biosanitaria de Granada (ibs.GRANADA), 18012 Granada, Spain; 4 Servicio de Reproducción Asistida, Hospital Universitario de Torrecárdenas, 04009 Almería, Spain; 5 Health in Code S.L., 15008 A Coruña, Spain; 6 Departamento Bioquímica, Biología Molecular e Inmunología III, University of Granada, 18071 Granada, Spain; 7 Unidad de Enfermedades Infecciosas, Hospital Universitario Virgen de las Nieves, 18014 Granada, Spain

**Keywords:** CVID, immunodeficiency, gastrointestinal infections, *Giardia*, giardiasis, refractory, treatment, immunology, immunogenetic

## Abstract

Common variable immunodeficiency (CVID) is an antibody immunodeficiency with a wide variety of clinical and immunological manifestations, and whose genetic cause is found in about 25% of diagnosed cases. *Giardia lamblia* is one of the main causes of gastrointestinal infections in CVID. 5-Nitroimidazoles are the most used first-line treatment, but nitroimidazole-refractory giardiasis is increasing. Nevertheless, only a few cases of refractory giardiasis in CVID have been reported. This study aimed to determine the incidence of *Giardia* infection in our CVID cohort, shows our management experience and describes patients’ phenotypic features. Clinical data collection, immunological, immunogenetics and microbiology assays were performed, and previous cases of giardiasis in CVID were reviewed. The incidence of symptomatic giardiasis was 12.9%. The main immunological features were undetectable or decreased IgA levels and reduced switched memory B cells. A probable *PTEN* pathogenic variant was detected in one. Three patients responded to metronidazole but suffered reinfections, and one was a refractory giardiasis eradicated with innovative quinacrine plus paromomycin combination. This work could contribute to the decision-making and therapeutic management of future patients with CVID and giardiasis, highlighting the importance of the early detection and treatment of infections in patients with CVID to ensure a good quality of life.

## 1. Introduction

Common variable immunodeficiency (CVID) is the most prevalent symptomatic primary immunodeficiency (PID) in humans [1] and is included in the antibody predominant immunodeficiency category according to the International Union of Immunological Societies (IUIS) classification [2].

CVID is considered a complex group of PID due to its clinical and immunological heterogeneity, and the underlying genetic cause is mostly unknown. Genetic defects are detected in approximately 25% of the cases, involving defects in humoral and cell-mediated immunity [3,4]. Diagnostic criteria for CVID, according to the European Society for Immunodeficiencies (ESID), include a decrease in IgG (at least two standard deviations below the mean for age) and a marked decrease in at least one of the isotypes IgM or IgA, an impaired antibody production to vaccination or low percentage of switched memory B cells (<70% of age-related normal value), clinical manifestations of recurrent infections, autoimmune diseases or lymphoproliferation, the onset of clinical immunodeficiency at more than two years of age and the exclusion of other causes of hypogammaglobulinemia [5]. The defect in plasma cell differentiation causes hypogammaglobulinemia and abnormalities of circulating B cell subsets, with a normal or low absolute count of B cells [6]. Although profound T cell defects are not detected, alterations in their frequency and function can be found [7].

**Figure 1:**
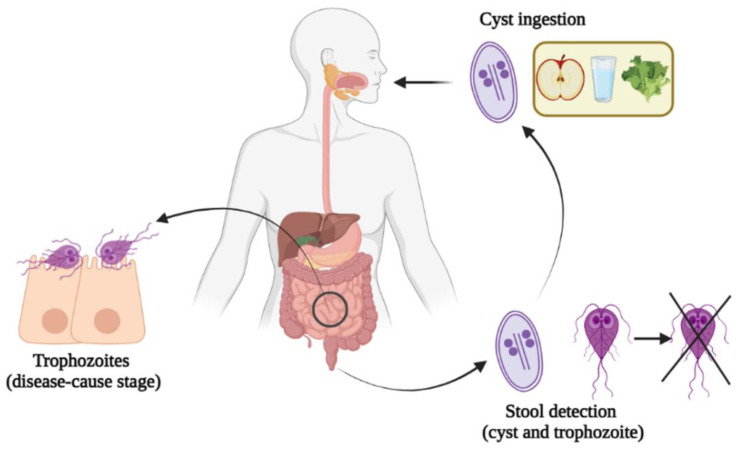
Lifecycle of *Giardia lamblia*. Infectious cysts are ingested via contaminated food or water, or by direct ingestion. In the human gastrointestinal tract, cysts excyst to release trophozoites, which cause disease, in part, by promoting the disruption of the intestinal epithelial barrier. Both the cysts and trophozoites can be detected in the stool, although the trophozoites released do not survive long.

Severe and recurrent infections are the clinical hallmark in CVID patients. *Giardia lamblia* is the most commonly identified gastrointestinal pathogen in CVID, followed by *Campylobacter jejuni* and *Salmonella species* [8]. *Giardia lamblia* (also termed *G. duodenalis* or *G. intestinalis*) is a flagellated parasitic protozoan with a lifecycle divided into two phases: the dormant infectious cyst and the proliferating trophozoite [9] (Figure 1). Clinical manifestations of *Giardia* infection are diverse, ranging from asymptomatic cases to diarrhea, abdominal pain, nausea, anemia, malabsorption, or weight loss. Classic diagnosis is performed by microscopic detection of trophozoites or cysts in stool samples, but in recent years, rapid immunochromatographic antigen tests and more sensitive real-time polymerase chain reaction (PCR) panels have appeared [10].

5-Nitroimidazole compounds, such as tinidazole or metronidazole, are the most common first-line treatment for *Giardia* infection [11]. Nitroimidazoles are usually also effective in CVID patients. However, CVID patients have a higher risk of chronification, reinfection and relapse rate due to their immunodeficiency status or malabsorption syndrome, and often require prolonged treatment [12,13].

Nitroimidazole-refractory giardiasis is increasing in the general population, linked with parasite drug resistance and host factors [14,15,16]. Nevertheless, few cases of refractory giardiasis in CVID patients have been published to date [17]. Currently, resistance *Giardia* detection is not possible to perform in most laboratories, and there is no standard treatment for refractory giardiasis. Empirical treatments are currently used, highlighting the use of a nitroimidazole other than metronidazole in monotherapy or with another drug, or other agents such as quinacrine or paromomycin [18].

The aim of this study was to determine the incidence of *Giardia* infection in patients diagnosed with CVID at our hospital center, describing our management experience and their demographic, clinical, immunogenetic, and immunological characteristics. In addition, we have performed a literature review of previous reports of *Giardia* infection in CVID patients.

## 2. Materials and Methods

### 2.1. Subjects of Study

Patients diagnosed with CVID and *Giardia lamblia* infection in the University Hospital Virgen de las Nieves between 2000 and 2021 were recruited for this study. The diagnosis of CVID was established based on the ESID criteria [5], excluding patients with other types of antibody immunodeficiencies, secondary antibody deficiencies, and T-cell deficiency.

*Giardia* infection was determined by stool *Giardia* antigen detection test, microscopy observation, molecular technique, or a combination of these, in patients with suggestive symptoms as described below. Refractory giardiasis was considered when *Giardia* persisted after one or more strategic treatments. We collected demographic and clinical data, family and personal history, and immunoglobulin levels at CVID diagnosis. Furthermore, we performed other immunological and immunogenetics assays during subsequent follow-ups of each patient. This study was reviewed and approved by the regional ethics committee (Portal de Ética de la Investigación Biomédica de Andalucía, PEIBA, code: 1206-N-22). Patients or their legal representatives provided their written informed consent to participate.

### 2.2. Immunological Evaluation

Serum immunoglobulins (Ig) levels (IgG, IgA and IgM) were measured by immunoturbidimetry using the automatic analyzer Alinity c system (Abbott Laboratories, Chicago, IL, USA). For cellular evaluation, EDTA whole blood samples were collected. Lymphocyte subpopulations (CD4+ T, CD8+ T, B and NK cells) were performed using BD Trucount tubes and the BD Multitest 6 Color BTNK kit (BD Biosciences, San Diego, CA, USA), which included the following mixtures of fluorophore-conjugated monoclonal antibodies (mAb): anti-CD45-PerCP-Cy5.5, anti-CD3-FITC, anti-CD8-APC-Cy7, anti-CD4-PE-Cy7, anti-CD19-APC, and anti-CD16+CD56-PE. B cell phenotype was performed with an eight-color panel of the following mAb: anti-CD45-APC-H7, anti-CD19-V500, anti-CD10-V450, anti-CD38-PE-Cy7, anti-CD21-PE, anti-CD27-PerCP-Cy5, anti-IgD-FITC, and anti-IgM-APC (BD Biosciences, San Diego, CA, USA), following EURO-Class classification. Cells were acquired on a BD FACSCanto II Flow Cytometer (BD Biosciences, San Diego, CA, USA), and the InfinicytTM22.0 software was employed for multiparametric analysis (Cytognos SL, Salamanca, Spain).

### 2.3. Immunogenetics

High-resolution genotyping of Human Leukocyte Antigen (HLA) class I (A, B and C) and II (DRB1 and DQB1) loci was performed using the LABType sequence-specific oligonucleotide typing test (One Lambda, Canoga Park, CA, USA). DNA whole blood isolation was carried out with the QIAMP DNA Blood Mini Kit, following the manufacturer’s instructions (Qiagen, Hilden, Germany). Target DNA was amplified by PCR using sequence-specific primers, followed by hybridization with allele-specific oligodeoxynucleotides coupled with fluorescent phycoerythrin-labelled microspheres. Fluorescence intensity was determined using a LABScan 100 system (Luminex xMAP, Austin, TX, USA). HLA alleles were assigned using the HLA-Fusion software (One Lambda, Canoga Park, CA, USA).

We also performed a clinical exome analysis based on Next-Generation Sequencing (NGS) that covers the coding regions of 4490 genes with clinical significance (SOPHiA Clinical Exome Solution, Lausanne, Switzerland), and analyzed 237 genes associated with primary immunodeficiencies (Supplementary Material S1). Sequencing was carried out on the NextSeq 1000 platform (Illumina, San Diego, CA, USA), and the results were analyzed with DDM v.5.8.0.3 program of Sophia Genetics and the IGV informatic application (Integrative Genomics Viewer). The reference genome sequence used in the alignment phase corresponds to the GRCh37/hg19 (UCSC) version. Bioinformatic predictors (MutationTaster and CADD) were used to evaluate the pathogenicity of the variants found. Genetic variants found were confirmed by Sanger sequencing.

### 2.4. Microbiology Giardia Infection Diagnosis

*Giardia* infection was diagnosed using antigen detection by immunochromatography (Rida Quick Cryptosporidium/Giardia/Entamoeba, R-Biopharm AG, Darmstadt, Germany), by microscopic observation of cysts in stool samples or by molecular diagnosis (FilmArray Gastrointestinal Panel, bioMérieux, Marcy l’Étoile, France).

### 2.5. Systematic Literature Review

**Figure 2:**
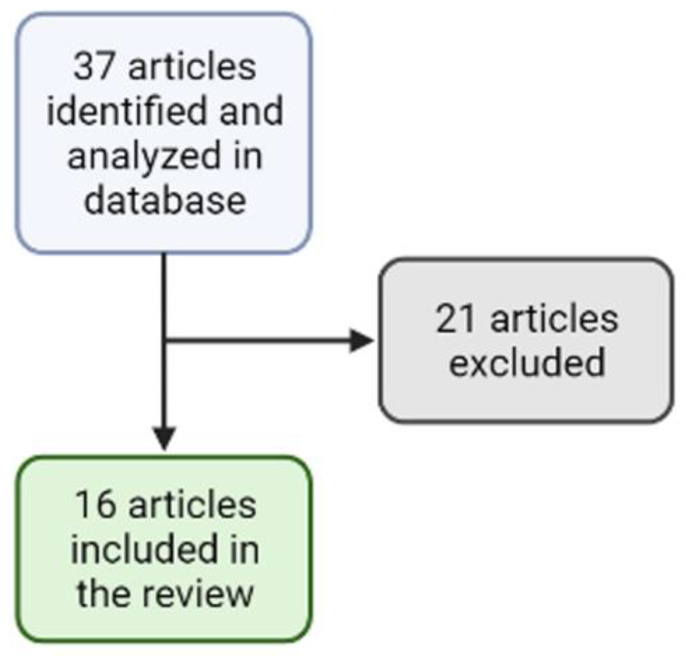
Flowchart of study selection for the narrative review.

A search was performed on the PubMed database up to 2022. Search terms used were: “giardia” OR “giardiasis”, AND “common variable immunodeficiency” (37 results). Articles available in English and Spanish were included, and articles that were not related to the subject of the study or did not provide sufficient data on treatment for giardiasis, microbiological and immunological diagnosis were excluded. Finally, 16 articles were included (Figure 2).

## 3. Results

### 3.1. Demographic Data and Clinical Manifestations

Thirty-one patients were diagnosed with CVID and treated with immunoglobulin replacement in our hospital center between 2000 and 2021. Four CVID patients (12.9%) suffered from *Giardia* infection during their clinical course. The median age of CVID patients with *Giardia* infection was 44 years old (28–55), two were male (50%) and two were female (50%). The median age at the time of CVID diagnosis was 33 (19–49) years. *Giardia* infection was detected between the first and the fourth year after beginning intravenous immunoglobulin (IVIG) treatment. CVID patients with *Giardia* infection had variable gastrointestinal symptoms and others such as asthenia or febricula. Three of them had *Giardia* reinfections (Cases 1, 2 and 4) and one was a refractory giardiasis (Case 3). In the two females, nodular intestinal lymphoid hyperplasia (NILH) was detected by endoscopy after the first *Giardia* infection, and Case 4 developed a Crohn’s disease-*like.* During the evolution of the CVID, without overlapping with the *Giardia* infection, patients also suffered from other clinical conditions, infections and comorbidities, which are summarized in Table 1.

Currently, Cases 1, 2 and 3 are clinically stable. Case 4 continues with gastrointestinal symptoms, arthritis, asthenia, anorexia and intense migraines, numbness in the face and loss of vision, probably associated with her Crohn-like disease. All CVID patients are with IVIG treatment. They receive 0,4 g/kg/day every 21 days except Case 4, which received 0,6 g/kg/day every 21 days because of her clinical condition.

### 3.2. Immunological Evaluation

The four CVID patients had undetectable or very low IgM and IgA levels from diagnosis. After IVIG treatment, all patients reached a normal IgG level, which is currently maintained. The analysis of lymphocyte subsets showed CD4+ T cell lymphopenia in Cases 3 and 4, a remarkable reduction of NK cells in Cases 1 and 4, and a very low B cell count in Case 1. B cell immunophenotype highlighted the reduction in switched memory B cells in all cases except in Case 3, in which they were absent. CD21*low* B cells were increased in Case 1 and transitional B cells were remarkably increased in Case 3. EURO-Class classification group and immunological data are indicated in Table 1.

**Table 1:** Epidemiological, clinical and immunological characteristics of patients with *Giardia* infection and CVID.

	Case 1	Case 2	Case 3	Case 4	Reference Values
Sex	Male	Male	Female	Female	
Age (years)	28	55	47	41	
Age at CVID diagnosis (years)	26	49	38	19	
Manifestation of *Giardia* infection	Diarrhea, weight loss	Diarrhea, weight loss, abdominal pain, malabsorption, rectal tenesmus, anal itching, asthenia, febricula	Diarrhea, weight loss, abdominal pain, rectal tenesmus, iron deficiency, anemia	Diarrhea, weight loss, abdominal pain, malabsorption, asthenia, iron deficiency	
Clinical and comorbid conditions	Recurrent respiratory infections, SARS-CoV-2 infection, psoriasis	Recurrent respiratory infection, otitis, *Campylobacter jejuni* infection, latent tuberculosis, verrucous cutaneous squamous cell carcinoma	Recurrent respiratory infections, NILH, ulcerative colitis, primary hypothyroidism, chronic hepatopathy, splenomegaly	Recurrent respiratory infections, bronchiectasis, HBV, SARS-CoV-2 infection, NILH, Crohn-*like* disease, arthralgias and arthritis	
*Immunoglobulins (Ig) at diagnosis*					
IgG (mg/dL)	164	444	461	200	540–1822
IgM (mg/dL)	<5	19	9	<5	22–240
IgA (mg/dL)	<5	10	<5	<5	70–400
*Immunoglobulins (Ig) after IVIG treatment*					
IgG (mg/dL)	1072	926	824	981	540–1822
IgM (mg/dL)	<5	19	<5	<5	22–240
IgA (mg/dL)	<5	<5	<5	<5	70–400
*Lymphocyte subsets* (cells/μL/%)					
CD3+	1441 (92)	1374 (69)	979 (60)	936 (81)	960–2600/61–84
CD3+CD4+	631 (40)	725 (36)	526 (32)	449 (39)	540–1660/32–60
CD3+CD8+	675 (43)	625 (31)	396 (24)	436 (38)	270–930/13–40
CD19+	28 (2)	356 (18)	115 (7)	127 (11)	122–632/6–27
CD3-CD56+CD16+	75 (4.8)	242 (12.1)	522 (32)	79 (6.9)	127–509/10.1–20.9
Ratio CD4/CD8	0.93	1.16	1.33	1.03	0.9–4.5
*B cell subsets (%)*					
CD19+ naive (IgD+CD27-)	46.2	78.3	24	75	53–86
CD19+ pre-switched memory (IgD+CD27+)	36.4	6.7	33.3	13.1	3.3–12.8
CD19+ switched memory (IgD-CD27+)	1.8	3.2	0	0.9	4–22
CD19+ CD21*low* (CD38*low*, IgM+)	10.2	2.2	0.3	1.5	0.4–4.5
CD19+ transitional (CD38*high*, IgM+)	0.4	0.7	34.5	0.6	0.9–6.3
EURO-Class classification group	smB-Tr^norm^ smB-21^lo^	smB+21^norm^	smB-Tr^hi^ smB-21^norm^	smB-Tr^norm^ smB-21^norm^	

NILH: nodular intestinal lymphoid hyperplasia; HBV: hepatitis B virus; Ig: immunoglobulin; IVIG: intravenous immunoglobulin.

### 3.3. Immunogenetics

HLA class I and II alleles genomic typing were performed and are shown in Supplementary Tables, along with HLA allele frequencies in the Spanish Caucasian population [19]. Case 3 had homozygosity in HLA-DQB1 alleles and Case 1 in HLA-DRB1. Case 1 had the ancestral haplotype 44.3.

The exome analysis performed was negative for Cases 1 and 3. Case 2 showed the heterozygous c.1555A>G (Lys519Glu) variant in the exon 17 of *TCF3* or *E2A* gen, which affects a region not associated with any of the major domains of the transcription factor it encodes. In silico and phylogenetic studies suggested that the affected residue is highly conserved and bioinformatic predictors did not give conclusive results on its pathogenicity. The amino acid substitution slightly modifies the physical–chemical properties of the protein. Databases consulted showed that there are asymptomatic heterozygous carriers in the general population as well as in asymptomatic carriers in families with severe agammaglobulinemia caused by biallelic variants (gnomAD frequency of 0.016%). Case 4 had the heterozygous c.1093G>A (Val365Ile) variant in exon 9 of phosphatase and tensin homolog (*PTEN*) gene, which affected the C-terminal domain of the mature protein phosphatase. In silico and phylogenetic studies suggest that the affected residue is highly conserved among vertebrate species and bioinformatic predictors showed that could be a pathogenic variant (MutationTaster score: 1, DANN score: 0.969). The amino acid substitution slightly modifies the physical–chemical properties of the protein. The database consulted points to a rare variant (gnomAD frequency < 0.01%), only present in two heterozygous carriers from the European population (Supplementary Material S2).

### 3.4. Giardia Infection Diagnosis and Treatment

The first *Giardia* infection was diagnosed by the *Giardia*/*Cryptosporidium* antigen test in Cases 1 and 2. Case 3 was diagnosed by an antigen test and multiplex PCR and Case 4 by cysts stool detection. The detection of reinfection, treatment failure and eradication of *Giardia* were performed by (a) stool cultures and/or the antigen test repetition in Case 2, (b) an antigen test and a multiplex PCR with symptom remission in Case 3, and (c) the remission of symptoms and negative antigen test in Case 4. In Case 1, it was not possible to assure *Giardia* eradication after the first infection because microbiological tests were not performed and the diagnosis was based on the remission of symptoms. In the reinfection, a stool examination was performed and *Giardia* cysts were not detected, confirming its eradication. None of the patients currently have evidence of *Giardia* infection, with antigen test negative.

First-line *Giardia* infection treatment was metronidazole, which successfully cured infection in Cases 1, 2 and 4. Quinacrine plus paromomycin was the successful treatment used for refractory giardiasis in Case 3 (Table 2).

### 3.5. Database Review Results

We found 17 published cases of giardiasis in CVID. The median age was 39 years old (15–62), eleven were male (64.7%) and six were female (35.3%). Six were refractory giardiasis (35.3%). The main clinical manifestations were diarrhea (14/17; 82.3%), weight loss and abdominal symptoms (9/17; 52.9%), and three patients had splenomegaly (3/17; 17.6%). All CVID patients showed undetectable or decreased IgA levels. All summarized data are collected in Table 3.

## 4. Discussion

The incidence of symptomatic giardiasis in our CVID cohort was 12.9%. The main clinical manifestations of *Giardia* infection were diarrhea, weight loss and abdominal pain, both in our patients and in review patients. It is important to make a correct differential diagnosis with other entities such as celiac disease and inflammatory bowel disease (IBD), which was performed in our patients, because gastrointestinal symptoms are very common in CVID, especially transient or persistent diarrhea [35]. Gastrointestinal *Giardia* symptoms in Cases 2 and 4 produced protein loss and malabsorption, which made it difficult to maintain their IgG levels in the normal range despite IVIG treatment. Immunoglobulin replacement therapy is the basic treatment of these patients, which improve their symptoms and their quality of life, but also prophylactic and therapeutic antibiotics for their recurrent infections [8]. Antibiotics stimulate intestinal dysbiosis, producing digestive alterations and favoring chronic or refractory infections by gastrointestinal pathogens such as *Giardia*. The gut microbiome has been shown to play a key role in determining susceptibility or resistance to *Giardia* colonization by modulating immune responses, while the parasite itself can influence the immune response to the host [36]. Recent research has recognized giardiasis as an important risk factor for developing long-term postinfectious syndromes, such as IBD, chronic fatigue syndrome, and extraintestinal consequences such as arthritis or allergies, even months or years after parasite clearance [16,37], which could be one of the reasons for the Crohn’s disease-*like* symptomatology in Case 4.

It should be noted that Cases 3 and 4 had nodular intestinal lymphoid hyperplasia (NILH), which is also found in CVID patients evaluated in the literature review (6/17; 35.3%). NILH is a rare condition that can occur as a form of compensation for the functionally inadequate intestinal lymphoid tissue found in patients with immunodeficiencies. However, NILH has also been associated with *Giardia lamblia* infection, and in some cases, its eradication has resulted in NILH reduction [23], or with an overregulation of the response mechanisms of the lymphoid tissue associated with the digestive tract [29]. In Cases 3 and 4, neither possibility can be ruled out, but it would be interesting to monitor its evolution because NILH could represent an intermediate stage and a risk factor for the development of lymphoma [38].

IgA, Th17 and CD4+ T cells are key in the immune response against *Giardia* [39]. Undetectable or very low levels of IgA are a characteristic in our four patients and in all available review patients, an immunoglobulin that is not replaced with IVIG treatment and that is key in the defense of the intestinal mucosa. Various studies have associated its deficiency with a greater capacity for binding and proliferation of foreign pathogens such as *Giardia* to the intestinal epithelium [12]. T CD4+ lymphopenia found in Case 3 could be another immunological factor resulting in refractory giardiasis. The remarkable reduction of B cells in Case 1 led us to suspect Bruton’s disease but it was ruled out by the NGS study. A remarkable NK cell lymphopenia was detected in Cases 1 and 4. These cells are important in the antiviral and antitumor defense. However, in CVID patients, NK cells have been associated with high frequencies of severe bacterial infections and non-infectious complications, suggesting that NK cells also have a role in controlling bacterial infections [40].

The EURO-Class classification was a multicenter European effort that allowed defining different subgroups of patients with CVID based on their B cell phenotype and some clinical features [6]. In our study, all cases had a reduction in switched memory B cells. The increase in CD21*low* cells is the strongest marker associated with splenomegaly. Case 3 had splenomegaly, but CD21*low* B cells were not altered, and she had also a remarkable increase of transitional B cells, without lymphadenopathy. In one literature case with refractory giardiasis, lymphadenopathy and splenomegaly were detected, but both regressed after *Giardia* eradication [26].

Different genetic variants have been involved in the pathogenesis of CVID, but most patients do not have a specific genetic defect background [41]. The NGS analysis detected possible CVID-associated variants in two of our patients. In Case 2, a heterozygous variant in *TCF3* gene (p.Lys519Glu) was detected. This gene is located on chromosome 19p13.3 and codes for transcription factors that promote the expression of genes involved in lymphopoiesis, differentiation and maturation of B and T cells. This variant has not been previously described but its presence in the control population and in the asymptomatic carriers of families with severe agammaglobulinemia caused by biallelic variants, as well as the contradictory results of in silico predictors, point to a benign variant [42,43]. In Case 4, a heterozygous variant in *PTEN* gene (p.Val365Ile) was detected. This gene is located on chromosome 10q23.31 and codes for a phosphatase that acts as a tumor suppressor gene. This enzyme also participates in the PI3K/Akt signaling pathway and, thus, in the differentiation and homeostasis of T and B cells. It has previously been considered a variant of uncertain significance (VUS) according to human databases in patients with Cowden syndrome type 1, a condition within the group of syndromes related to the development of hamartomas (PHTS) following an autosomal dominant pattern of inheritance [44]. These patients had heterogeneous phenotypes, some of whom had PHTS with a CVID phenotype, exhibiting a decrease in switched memory B cells and a reduction in functional and mature NK cells, immunological alterations that are present in our Case 4 patient. Likewise, loss-of-function mutations in *PTEN* have also been described as being associated with activated phosphoinositide-3-kinase delta syndrome-*like* (APDS-*like*) because it acts as an antagonist in the PI3K-delta signaling pathway [45,46]. Cosegregation and functional studies will be necessary to confirm the pathogenicity of this variant in the context of CVID, which will be a future objective of our group.

The HLA class I and II genes code for cell surface molecules specialized in antigen presentation to T cells and play a key role in the immune response. Previous studies have reported a positive association between certain HLA alleles and different diseases, including susceptibility to CVID [47]. It has been described that the extended haplotype HLA-A1, -B8, -DR3 is more frequent in these patients [48] and that homozygosity in the HLA class II region, specially HLA-DQ, is associated with greater susceptibility to CVID [49]. Curiously, Case 3 patient has homozygosity in HLA class II, our giardiasis refractory case. This homozygosity could result in a lower repertoire of HLA class II molecules on the cell surface, resulting in a lower capacity to respond to foreign or pathogenic antigens and contributed to CVID susceptibility to specific environmental conditions. Moreover, Case 4 has the HLA-DRB1*13:01 allele, which has been previously associated with susceptibility to *Giardia* infection [50]. On the other hand, Case 1 patient has the C*06:02 allele, which is one of the most strongly HLA alleles associated with psoriasis susceptibility [51]. None of the four patients presented the ancestral haplotype 8.1 (HLA-A1, -B8, -DR3), although Case 1 had the ancestral haplotype 44.3 (A*29:02; B*44:03; C*16:01; DRB1*07:01; DQB1*02:02). It would be advisable to extend the study to a bigger CVID cohort to have more precise data on this evidence.

First-line giardiasis treatment is based on the use of nitroimidazoles [11]. In our cohort, 75% (3/4) responded to metronidazole, evidenced by negative microbiological tests and remission of symptoms. This is similar to the case reports included in the literature review, although reinfections occurred in all of our cases. However, one of the highlights of this work is the first refractory giardiasis case diagnosis in our hospital in a CVID patient (Case 4) and, to our knowledge, the first time that the combination of quinacrine plus paromomycin has been described and has been effective. We consider the treatment successful because stool examination has remained negative to date. So far, only six refractory giardiasis cases in CVID patients have been published, and different curative treatments have been applied [20,22,26,30,31,33]. Currently, there is no standard therapy for refractory giardiasis, and multiple drugs have been tried [15]. Tinidazole, a derivative of metronidazole, has been shown to be more effective than metronidazole [11,39] but was not effective in Case 3. The combination of metronidazole and albendazole, the latter a benzimidazole, is more effective in treating refractory disease than albendazole alone [15], but again no response was obtained as a third-line treatment. On the other hand, the efficacy and safety of the use of quinacrine in refractory giardiasis have been demonstrated in various studies, mainly in monotherapy or combined with metronidazole [22,52,53]. Quinacrine, also known as mepacrine, was the first antimalarial drug used to treat giardiasis, but its use was limited by detecting possible adverse effects of psychosis. Despite everything, it is usually well-tolerated and has a clinical efficacy of >90% [54]. In the case of paromomycin, its activity is variable against *Giardia* (55–90%) and is rarely used. It can be employed in cases of resistance or pregnancy because it has a low absorption spectrum and does not have systemic effects [14]. It is usually well-tolerated, although being an aminoglycoside, it can cause nephrotoxicity and ototoxicity [15,54]. The quinacrine and paromomycin combination therapy should be used in a larger patient cohort to confirm its efficacy and safety, and more clinical trials are needed to establish the optimal therapy for patients with refractory giardiasis.

*Giardia* drug resistance is currently undetectable in most microbiology laboratories. This is due, on the one hand, because the parasite culture is difficult and takes a long time, and success rates are relatively low [15] and, on the other hand, because resistance depends more on epigenetic factors and post-translational modifications than on genetic variants in the parasite genome. For these reasons, it is not yet possible to develop a routine microbiology technique to detect *Giardia* susceptibility [14]. Moreover, since there are few cases of refractory giardiasis, multicenter studies are required to define the best therapeutic alternatives. At the diagnostic level, it would be interesting to develop a strategy that combines the culture of the parasite with sequencing and comparative proteomics [55], which may allow moving from empirical treatment to targeted treatment in the future.

The early detection and treatment of infections in patients with CVID are crucial to ensure a good quality of life. Moreover, the immunological and genetic characterization of these patients is necessary to improve their clinical follow-up and to better understand the pathogenesis of CVID. One limitation of this work was that the cohort of CVID patients who suffered from symptomatic *Giardia* infection was small. Still, to our knowledge, we present a probably pathogenic *PTEN* variant associated with the CVID phenotype, and, for the first time, a case of refractory giardiasis in CVID that was successfully treated with quinacrine plus paromomycin combination. These data could contribute to the decision-making and therapeutic management of future patients with CVID and giardiasis, especially in refractory giardiasis cases.

## Figures and Tables

**Table 2:** *Giardia* infection treatments in each patient.

	Case 1	Case 2	Case 3	Case 4
First-line treatment	Metronidazole 250 mg/8 h for 7 days	Metronidazole 250 mg/8 h every 5 days	Metronidazole 500 mg every 8 h for 7 days	Metronidazole 250 mg every 8 h for 8 days
Other treatments	Reinfection: Metronidazole 250 mg/8 h for 20 days	Reinfection: Metronidazole 500 mg every 8 h for 10 days	1^st^ failure: Metronidazole 500 mg every 8 h for 14 days. 2^nd^ failure: Tinidazole 2 g spread over two consecutive days. 3^rd^ failure: Metronidazole plus Albendazole (400 mg/12 h) for 10 days. 4^th^ treatment: Quinacrine 100 mg plus paromomycin 400 mg every 8 h	Reinfection: Metronidazole 500 mg every 8 h for 7 days
Successful *Giardia* infection drug	Metronidazole	Metronidazole	Quinacrine + Paromomycin	Metronidazole

mg: milligrams; g: grams; h: hours.

**Table 3:** Cases of giardiasis in CVID documented in the literature.

References	Age (Years), Gender	Giardiasis	Clinical Giardiasis Manifestations	IgA Level	Curative Treatment	Microbiological Cure	Other Characteristics
Taylor GC et al., 1987 [20]	47, Male	Refractory giardiasis	Diarrhea, abdominal cramps, weight loss	Undetectable	Metronidazole + Quinacrine	Confirmed by stool microscopy	Failure treatment: Metronidazole
Bästlein C, Burlefinger R et al., 1988 [21]	31, Male	Chronic giardiasis	Abdominal pain, splenomegaly	Decreased	Metronidazole	Not done (symptomatic relief)	NILH
Nash TE et al., 2001 [22]	46, Female	Refractory giardiasis	Nausea, diarrhea, pernicious anemia,	N.A.	Metronidazole + Quinacrine	Confirmed by *Giardia* antigen test	Failure treatments: Metronidazole
De Weerth, et al., 2002 [23]	40, Female	Giardiasis	Diarrhea, abdominal pain, weight loss	Undetectable	Metronidazole	Not done (symptomatic relief)	NILH detection, which reduces after *Giardia* eradication
Onbaşi K, Günşar F et al., 2005 [24]	39, Female	Chronic giardiasis	Diarrhea, weight loss	Decreased	Metronidazole	Not done (symptomatic relief)	
Ogershok PR, Hogan MB et al., 2006 [25]	24, Male	Giardiasis	Diarrhea	Undetectable	N.A,	N.A.	
	15, Male						
Ramsey NC et al., 2010 [26]	46, Male	Refractory giardiasis	Abdominal pain, diarrhea, splenomegaly and lymphadenopathy	N.A.	Tinidazole + Paromomycin + Bacitracin + Cholestyramine	Confirmed by *Giardia* antigen test (EIA)	Lymphadenopathy and splenomegaly regressed after *Giardia* eradication. Failure treatments: metronidazole, cholestyramine + tinidazole
Domínguez-López ME, González-molero I, et al., 2011 [27]	49, Male	Chronic giardiasis	Diarrhea, weight loss	N.A.	N.A.	N.A.	NILH, gastrectomy for gastric cancer
Silva GB et al., 2012 [28]	62, Female	Giardiasis	Diarrhea, weight loss, abdominal pain, and intermittent fever	Decreased	Metronidazole	Not done (symptomatic relief)	*Isospora belli* coinfection
Olmez S, Aslan M et al., 2014 [29]	38, Male	Giardiasis	Dyspeptic complaints	Decreased	N.A.	Not indicated	NILH detection
Choi JH et al., 2017 [30]	41, Female	Refractory giardiasis	Intermittent diarrhea	Undetectable	Metronidazole + Albendazole	Confirmed by stool microscopy	NILH detection, which reduces after *Giardia* eradication
							Failure treatments: metronidazole, albendazole, tinidazole
Paranjpe SM et al., 2017 [13]	18, Male	Chronic giardiasis	Diarrhea, weight loss, abdominal pain	Remarkably decreased	Metronidazole	Not done (symptomatic relief)	
Atalaia-Martins C, Barbeiro S et al., 2017 [31]	42, Female	Refractory giardiasis	Epigastric discomfort, postprandial fullness, diarrhea, weight loss, fatigue, anemia	Undetectable	N.A.	Not indicated	*Helicobacter pylori* coinfection, gastric dysplasia, NILH. Failure treatments: metronidazole, tinidazole, albendazole
Saurabh K, Nag VL et al., 2017 [32]	16, Male	Giardiasis	Diarrhea, nausea, vomiting, pedal edema	Decreased	Nitazoxanide + Metronidazole	Confirmed by stool microscopy	*Hymenolepis nana* coinfection. Finally, he died because of his immunological condition.
Kaya F et al., 2018 [33]	28, Male	Refractory giardiasis	Diarrhea, nausea and bloating, abdominal cramps and weight loss	Decreased	N.A.	N.A.	Failed treatments: Metronidazole, ornidazole, albendazole, nitazoxanide, trimethoprim/sulfamethoxazole + metronidazole, nitazoxanide + paromomycin
Sousa D, 2020 [34]	33, Male	Chronic giardiasis	Diarrhea, weight loss, iron-deficiency anemia, splenomegaly	Undetectable	Metronidazole	N.A.	

N.A.: not available. EIA: enzyme immunoassay. NILH: nodular intestinal lymphoid hyperplasia.

## Supplementary Materials

The following supporting information can be downloaded at https://www.mdpi.com/article/10.3390/jcm11237007/s1, Supplementary Material 1: Genes associated with primary immunodeficiency analyzed by Next-Generation sequencing; Supplementary Tables S1 and S2: Human Leukocyte Antigen (HLA) genomic typing of CVID patients with giardiasis; Supplementary Material 2: Genetic results and sanger sequencing methodology..
