# Supplementary material for: Incidence, Management Experience and Characteristics of Patients with Giardiasis and Common Variable Immunodeficiency
Source: J Clin Med. 2022 Nov 27;11(23):7007. doi: 10.3390/jcm11237007 (PMC9740678; doi:10.3390/jcm11237007)
Supplement: Supplementary file 1 [file jcm-11-07007-s001.zip › Suplementary Material S1.pdf]

Supplementary Material S1. Genes associated with primary immunodeficiency analyzed by Next-Generation sequencing.

ACP5 ACTB ADA ADAM17 AICDA AIRE AK2 AP3B1 AP3D1 APOL1 ATM B2M BCL10 BLM BLNK BRCA1 BRCA2 BRIP1 BTK C1QA C1QB C1QC C1R C1S C3 C5 C6 C7 C8A C8B C9 CARD11 CARD14 CARD9 CASP10 CASP8 CCBE1 CD19 CD27 CD3D CD3E CD3G CD40 CD40LG CD46 CD55 CD59 CD79A CD79B CD81 CD8A CEBPE CFD CFH CFHR1 CFHR5 CFI CFP CFTR CHD7 CIITA CLCN7 CORO1A CSF2RA CSF3R CTC1 CTLA4 CTSC CXCR4 CYBA CYBB DCLRE1C DEF6 DKC1 DNASE1L3 DNASE2 DNMT3B DOCK8 ELANE ERCC4 EXTL3 FADD FANCA FANCB FANCC FANCD2 FANCE FANCF FANCI FANCL FANCM FCN3 FERMT1 FERMT3 FOXN1 FOXP3 FPR1 G6PC3 G6PD GATA2 GFI1 HAX1 ICOS IFIH1 IFNAR1 IFNAR2 IFNGR1 IFNGR2 IGLL1 IKBKB IKBKG IKZF1 IL10 IL10RA IL10RB IL12B IL12RB1 IL12RB2 IL17F IL17RA IL1RN IL21R IL23R IL2RA IL2RG IL36RN IL6R IL6ST IL7R IRAK4 IRF4 IRF8 ITCH ITGB2 ITK JAK3 KDM6A KMT2D KRAS LAMTOR2 LCK LIG1 LIG4 LPIN2 LRBA LYST MAGT1 MALT1 MASP2 MCM4 MEFV MOGS MSH6 MTHFD1 MVK MYD88 NBAS NCF2 NCF4 NCSTN NFKB2 NFKBIA NHEJ1 NLRP1 NLRP12 NLRP3 NOD2 NRAS OAS1 ORAI1 OSTM1 PALB2 PEPD PIK3CD PIK3R1 PLCG2 PMS2 PNP POLD1 POLE2 POLR3A PRF1 PRKDC PSENEN PSMB8 PSTPIP1 PTEN PTPRC RAB27A RAC2 RAD51 RAD51C RAG1 RAG2 RANBP2 RASGRP1 REL RFX5 RFXANK RFXAP RHOH RNASEH2A RNASEH2B RNASEH2C RNF168 SAMD9 SAMHD1 SEMA3E SERPING1 SH2D1A SH3BP2 SLC29A3 SLC35C1 SLC46A1 SLC7A7 SLX4 SMARCAL1 SNX10 SP110 SPINK5 SRP72 STAT1 STAT3 STAT5B STIM1 STK4 STX11 STXBP2 TAP2 TAZ TBK1 TBX1 TCF3 TCIRG1 TCN2 TERC TERT TFRC TGFB1 TGFBR1 TGFBR2 THBD TICAM1 TINF2 TIRAP TLR3 TMC6 TMC8 TMEM173 TNFRSF11A TNFRSF13B TNFRSF13C TNFRSF1A TNFRSF9 TNFSF11 TP53 TRAF3 TRAF3IP2 TREX1 TTC37 TYK2 UNC13D UNC93B1 UNG USB1 VPS13B WAS WIPF1 WRAP53 XIAP XRCC2 ZAP70 ZBTB24
