# Supplementary material for: Incidence, Management Experience and Characteristics of Patients with Giardiasis and Common Variable Immunodeficiency
Source: J Clin Med. 2022 Nov 27;11(23):7007. doi: 10.3390/jcm11237007 (PMC9740678; doi:10.3390/jcm11237007)

Supplementary Material S2. Genetic results and sanger sequencing methodology.

Table S1. Genomic variants found by NGS in Cases 2 and 4.

Case	Gen	Chr	Exon	c.DNA	Protein	Mutation type	VAF (%)	Depth of coverage
2	*TCF3*	19	17	c.1555A>G	p.(Lys519Glu)	missense	42.9%	338
4	*PTEN*	10	9	c.1093G>A	p.(Val365Ile)	missense	50.58%	259

Sanger sequencing methodology.

Sanger sequencing of variants in *TCF3* and *PTEN* genes were performed by bidirectional Sanger sequencing using specific primers targeted to the fragment sequence of interest referred to the hg37 genome reference. We designed specific primers to amplified the exon 9 of *PTEN* (Fw:CCTAGCAAGAAAGAAAATGTTGAA; Rv:AGTGTCAAAACCCTGTGGATG) and two couples of primers for sanger sequencing of the previous PCR product (Fw1:CCTAGCAAGAAAGAAAATGTTGAA;Fw2:CCTAGCAAGAAAGAAAATGTTGAA and Rv:AGGCCTCTTAAAGATCATGTTTGT). Regarding the variant in *TCF3*, we designed specific primers both valid for amplification and sequencing of exon 17 (Fw:CCTCACCACAGGAGCACAAA; Rv:CAGTGAGGTTGGGGGAAGAG).

Figure S1. The electropherogram showed the heterozygous c.1555A>G variant in the exon 17 of the *TCF3* gene in Case 2 (the R indicates the change). The superior orange line represents the sequence of the reference genome.

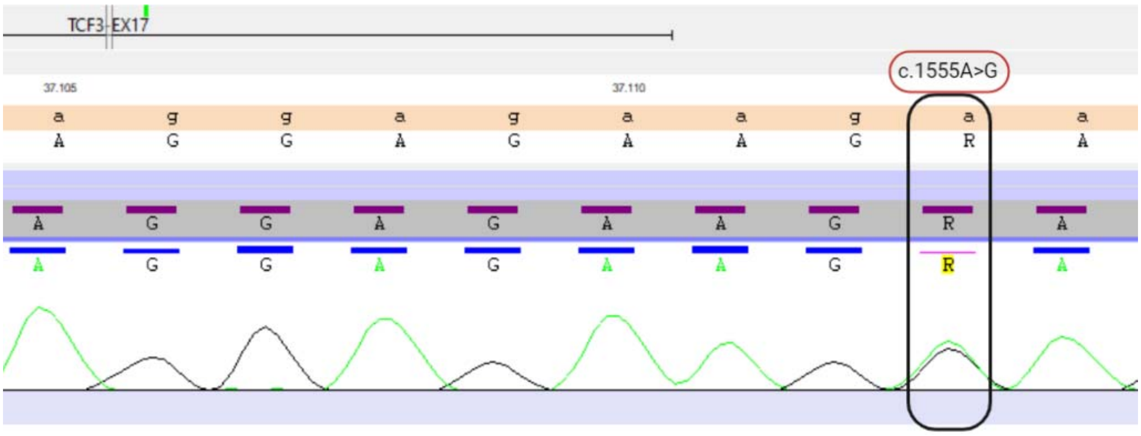

Figure S2. The electropherogram showed the heterozygous c.1093G>A variant in the exon 9 of the *PTEN* gene in Case 4 (the R indicates the change). The superior orange line represents the sequence of the reference genome.

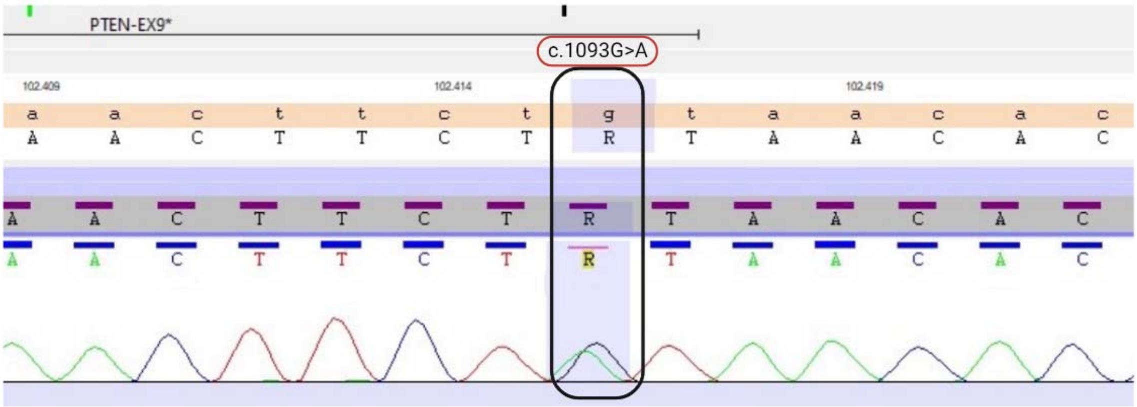

Supplement: Supplementary file 1 [file jcm-11-07007-s001.zip › Suplementary Material S2.pdf]
