# Supplementary material for: Incidence, Management Experience and Characteristics of Patients with Giardiasis and Common Variable Immunodeficiency
Source: J Clin Med. 2022 Nov 27;11(23):7007. doi: 10.3390/jcm11237007 (PMC9740678; doi:10.3390/jcm11237007)
Supplement: Supplementary file 1 [file jcm-11-07007-s001.zip › Suplementary Tables.pdf]

Supplementary Tables. Human Leukocyte Antigen (HLA) genomic typing of CVID patients with giardiasis.

Table S1. HLA class I (A, B and C loci) genomic typing of CVID patients with giardiasis. HLA allele frequencies in the Spanish Caucasian population are indicated (obtained from Gonzalez-Galarza FF et all., 2020).

	HLA-A	Allele Frequency	HLA-A	Allele Frequency	HLA-B	Allele Frequency	HLA-B	Allele Frequency	HLA-C	Allele Frequency	HLA-C	Allele Frequency
**Case 1**	A*01:01	0.0985	A*29:02	0.0783	B*44:03	0.0998	B*57:01	0.0234	C*06:02	0.0737	C*16:01	0.0807
**Case 2**	A*29:02	0.0783	A*32:01	0.0384	B*37:01	0.0107	B*51:01	0.0746	C*02:02	0.0509	C*06:02	0.0737
**Case 3**	A*02:01	0.2368	A*33:01	0.0191	B*14:02	0.0399	B*49:01	0.0371	C*07:01	0.1301	C*08:02	0.0555
**Case 4**	A*01:01	0.0985	A*03:01	0.1024	B*07:02	0.0799	B*38:01	0.0362	C*07:02	0.0907	C*12:03	0.0686

HLA: Human leukocyte antigen.

Table S2. HLA class II (DRB1 and DQB1 loci) genomic typing of CVID patients with giardiasis. HLA allele frequencies in the Spanish Caucasian population are indicated (obtained from Gonzalez-Galarza FF et all., 2020).

	HLA-DRB1	Allele Frequency	HLA-DRB1	Allele Frequency	HLA-DQB1	Allele Frequency	HLA-DQB1	Allele Frequency
**Case 1**	DRB1*07:01	0.1696	-		DQB1*02:02	0.1481	DQB1*03:03	0.0288
**Case 2**	DRB1*04:03	0.0158	DRB1*10:01	0.0140	DQB1*03:04	0.0022	DQB1*05:01	0.1283
**Case 3**	DRB1*11:02	0.0151	DRB1*11:04	0.0405	DQB1*03:01	0.1810	-	
**Case 4**	DRB1*11:01	0.0626	DRB1*13:01	0.0719	DQB1*03:01	0.1810	DQB1*06:03	0.0736

HLA: Human leukocyte antigen.
